# Chromosome 21 Scan in Down Syndrome Reveals DSCAM as a Predisposing Locus in Hirschsprung Disease

**DOI:** 10.1371/journal.pone.0062519

**Published:** 2013-05-06

**Authors:** Anne-Sophie Jannot, Anna Pelet, Alexandra Henrion-Caude, Asma Chaoui, Marine Masse-Morel, Stacey Arnold, Damien Sanlaville, Isabella Ceccherini, Salud Borrego, Robert M. W. Hofstra, Arnold Munnich, Nadège Bondurand, Aravinda Chakravarti, Françoise Clerget-Darpoux, Jeanne Amiel, Stanislas Lyonnet

**Affiliations:** 1 INSERM U-781, AP-HP Hôpital Necker-Enfants Malades, Paris, France; 2 Département de Génétique, Université Paris Descartes, Faculté de Médecine, Paris, France; 3 INSERM U-955, Créteil, France; 4 Center for Complex Disease Genomics, McKusick-Nathans Institute of Genetic Medicine, Johns Hopkins University School of Medicine, Baltimore, Maryland, United States of America; 5 HCL, Service de génétique, Bron, France; 6 INSERM U-1028, CNRS UMR5292, Université Claude Bernard Lyon 1, Equipe TIGER, Lyon, France; 7 Laboratorio di Genetica Molecolare, Istituto Gaslini, Genova, Italy; 8 Unidad de Gestión Clínica de Genética, Reproducción y Medicina Fetal, Instituto de Biomedicina de Sevilla (IBIS), Hospital Universitario Virgen del Rocío/CSIC/Universidad de Sevilla, Sevilla, Spain; 9 CIBER de Enfermedades Raras, ISCIII, Sevilla, Spain; 10 Department of Clinical Genetics, ErasmusMC, University of Rotterdam, Rotterdam, The Netherlands; Wadsworth Center, United States of America

## Abstract

Hirschsprung disease (HSCR) genetics is a paradigm for the study and understanding of multigenic disorders. Association between Down syndrome and HSCR suggests that genetic factors that predispose to HSCR map to chromosome 21. To identify these additional factors, we performed a dose-dependent association study on chromosome 21 in Down syndrome patients with HSCR. Assessing 10,895 SNPs in 26 Caucasian cases and their parents led to identify two associated SNPs (rs2837770 and rs8134673) at chromosome-wide level. Those SNPs, which were located in intron 3 of the *DSCAM* gene within a 19 kb-linkage disequilibrium block region were in complete association and are consistent with *DSCAM* expression during enteric nervous system development. We replicated the association of HSCR with this region in an independent sample of 220 non-syndromic HSCR Caucasian patients and their parents. At last, we provide the functional rationale to the involvement of DSCAM by network analysis and assessment of SOX10 regulation. Our results reveal the involvement of *DSCAM* as a HSCR susceptibility locus, both in Down syndrome and HSCR isolated cases. This study further ascertains the chromosome-scan dose-dependent methodology used herein as a mean to map the genetic bases of other sub-phenotypes both in Down syndrome and other aneuploidies.

## Introduction

Hirschsprung disease (HSCR, aganglionic megacolon) is the most frequent genetic cause of congenital intestinal obstruction. The *RET* gene, which maps on chromosome 10 (10q11.2) and encodes a tyrosine kinase receptor, is implicated in the vast majority of HSCR cases, both isolated and syndromic cases [1]. Patients harbor either mutations in the coding sequence [2], [3] or a non-coding polymorphism (rs2435357) in an enhancer element located in intron 1 leading to a decreased *RET* allele expression, which defines a hypomorphic allele [4].

Genetic factor(s) on chromosome 21 are suspected to increase HSCR susceptibility. First, Down syndrome (DS) is the most frequent (i.e >90%) chromosomal anomaly in HSCR and occurs in 2–10% of cases [2]. Down syndrome patients with HSCR (HSCR-DS) have no *RET* mutation in the coding sequence but a significantly increased frequency and over-transmission of a hypomorphic T allele in *RET* at rs2435357 SNP [1], [5], [6]. Two approaches have been used to identify genetic factors on chromosome 21 in these patients. The first one was to determine the shorter region of overlap (SRO) between segmental trisomy 21 and HSCR. This led to identify a region spanning 33.5–46.25 Mb on chromosome 21 [7]. The second approach consisted to analyze gene expression studies in the enteric nervous system of HSCR mouse models. This led to identify 9 genes mapping to the syntenic mouse DS critical region [8]. However, the involvement of these genes in HSCR was not confirmed in 62 patients with DS and HSCR [5]. Hitherto, no gene on chromosome 21 was demonstrated to increase their susceptibility to HSCR.

Here, we performed a chromosome scan to test for association with chromosome 21 on a series of Caucasian patients with DS and HSCR and their parents. A genome-wide scan on a cohort of isolated Caucasian HSCR cases and their parents was used for validation.

## Patients and Methods

### Patients

The chromosome-wide association sample, referred to as the HSCR-DS sample, consisted in 26 triads collected through the International Hirschsprung Disease Consortium in which the proband had both HSCR and Down syndrome. Among them, 12 were recruited in France, 8 in the USA, 4 in Spain, one in The Netherlands and one in Italy.

The validation sample consisted of DNA from 220 Caucasian cases with isolated HSCR and their parents, referred to as the isolated-HSCR sample, also collected through the International Hirschsprung Disease Consortium.

### Genotyping

An Affymetrix GeneChip Human Mapping 6.0 run in McKusick-Nathans Institute of Genetic Medicine, Johns Hopkins University School of Medicine, Baltimore, was available for the chromosome-wide association study.

Genotyping was performed using R 2.15.2 software by taking the following steps: (i) intensity extraction (3 intensities per SNP allele using packages pd.genomewide.6 and oligoClasses [9], [10]), (ii) normalization (i.e ratio of the mean intensity obtained for one of the SNP allele divided by the sum of all 6 intensities obtained for the SNP), and (iii) genotype calling. Genotype calling was performed using the K-means method for independent individuals (kmeans function). As this method does not use information on pedigree, the genotypes that are not possible under Mendelian law are also called. Therefore, we used an adaptation from the K-means method that incorporates the pedigree information by updating all members of a family together [11]. To do so, we applied the R code for the family-based genotype calling methods (SNPCaller, function mkmeans.tri) to our dataset. While either methods, whether K-means or its adaptation to family-based design, showed good performance in simulated datasets, the performance was better for the K-means method adapted to family-based design [11]. But, in order to apply the K-means method adapted for family-based design, we first called genotypes by the K-means method for independent individuals to infer the non-disjoining parent (NDJP) and the correctly disjoining parent (CDJP) as described below. Calling was performed by clustering all SNPs of a given trio rather than clustering all trios for each SNP, because the number of observations for each SNP was too low to cluster (e.g. in trisomic children, there were only 26 observations to infer 4 clusters). When calling genotypes using the K-means method for independent individuals, we infered the NDJP and the CDJP using the following properties:

- consider a SNP with allele A and B and (G1,G2,G3) a trio genotype with G1, G2 and G3 being respectively the father, mother and trisomic child genotype.
- if the father is the non-disjoining parent, then the following trio genotypes are not possible under Mendelian law: F1 = (AA,AB,ABB) and F2 = (BB,AB,AAB)
- if the mother is the non-disjoining parent, then the following trio genotypes are not possible under Mendelian law: M1 = (AB,AA,ABB) and M2 = (AB,BB,AAB)
- when genotypes are called with the K-means method for independent individuals, the configurations that are not possible under Mendelian law are not discarded. But these configurations should rarely be called. For instance, M1 and M2 configurations should rarely be called if the mother is the non-disjoining parent.
- as a consequence the NDJP should be the mother if the number of M1 and M2 trios among trios with heterozygotes mother is significantly greater than the number of F1 and F2 trios among trios with heterozygotes father; the NDJP should be the father if it is significantly lower.

Once the non-disjoining parent was defined, we called genotypes using the adaptation of the K-means method to family-based data. We discarded SNPs with more than 25% of discrepancies between the calls given by the two methods in children. We also checked for Hardy-Weinberg equilibrium in parents and discarded those SNPs with a p-value below 10^−4^.

For the isolated-HSCR sample, genotypes were extracted from the Affymetrix 500 K Array Set (250 K *NspI* and *StyI* array). 125 triads were run on the *NspI* array, 128 on the *StyI* array (33 on both arrays). Genotypes were called by the stand-alone command-line BRLMM (Bayesian Robust Linear Model with Mahalanobis distance) program [12]. SNPs with a MAF<5%, departing from HWE (p<0.05) or with a call rate below 80% were removed.

### Statistical Analysis

All analyses were performed using R 2.15.2 software. Due to the size-contraint of our trisomic sample, we chose an extension of the case/pseudo-control test to test the association between each SNP and HSCR. In contrast to a method based on numerical optimisation of maximum likelihood, the issue of parameter estimation does not appear with small sample size contrarely to the trisomic transmission disequilibrium test proposed as stated by Xu et al [13]. The extension of the case/pseudo-control test to trisomic sample is based on the following method.

Let assume a SNP with alleles A and a:

- X the number of A alleles in the mother genotype (X = 0, 1 or 2)
- Y the number of A alleles in the father genotype (Y = 0, 1 or 2)
- Z1 the number of A alleles in the child genotype (X = 0, 1, 2 or 3)
- Z2 the number of A alleles in the pseudo-control genotype (X = 0, 1, 2 or 3) determined by the following equations:
  - Z2 = 2X+Y−Z1 in the case of maternal non-disjunction
  - Z2 = X+2Y−Z1 in the case of paternal non-disjunction

In the case of no association, then we have Z1–Z2 = 0.

We therefore tested the hypothesis of no association for each SNP using a Wilcoxon paired test.

To illustrate the construction of pseudo-controls, suppose the mating type is AA×Aa where the Aa parent is the non-disjoining parent. Then the correctly disjoining parent must contribute to an A allele for both the case and the pseudo-control. The non-disjoining parent contributes Aa if the two chromosomes are not reduced to homozygosity. Therefore four gametes result from the meiosis: two diploid gametes Aa and two gametes with no chromosome. Two trisomic children could result from this couple, both with AAa genotypes, therefore both the transmitted and the untransmitted alleles are Aa and the case and the pseudo-control will have the same AAa genotype. If the two chromosomes of the non-disjoining parent are reduced to homozygosity, two diploid gametes could be formed by the non-disjoining parent: AA and aa. In this case, the case and pseudo-control genotypes would be AAA and Aaa respectively or the reverse.

For the isolated HSCR sample, we tested association using a Wilcoxon paired test comparing cases and their pseudo-controls.

Odds-ratios and corresponding 95% confidence interval were estimated using formulas proposed by Kazeem and Farrall [14].

To correct for multiple testing, we permuted cases and pseudo-controls status while keeping genotypes the same.

### Network Analysis

To analyze the biological involvement of the results, we also used an interactive and manually annotated database, which is derived from literature publications on proteins from the GWAS (MetaCoreTM, GeneGo, St Joseph, MI, USA). The GeneGo platform comprises signaling and metabolic pathways, which are manually curated. The database comprises approximately 700 representations of human and rodent signaling and metabolic pathways. The enrichment calculation uses the Fisher exact test or hypergeometric distribution to calculate the probability that the degree of overlap between the list of possible protein targets generated from the query compounded analysis and the protein represented in the functional ontology category can happen by chance, given an identical number of proteins selected at random from the protein universe annotated within the ontology.

### Analysis of SOX10 Binding Sites

Search for SOX10-binding sites was performed in silico using http://rvista.dcode.org/. Gel shift experiments were performed using truncated SOX10 versions (amino acids 1–188, 5 µg/reaction) and 0.5 ng of ^33^P-labeled probe A: 5′-GATCAATGCAGTGAAGTCAGTG**A**TAAGT-3′ and probe B: 5′-GATCAATGCAGTGAAGTCAGTG**G**TAAGT-3′ as previously described [15]. Probes containing one or two SOX10-binding sites from the *MITF* or *Cx32* promoter regions were used as controls (for sequences see [16], [17]). The two putative SOX10 binding sites identified are underlined in probe A.

### Ethics Statement

Ethic committee “Ile de France II” (Project AOM95224, P959892) approved the study protocol. Written informed consent was obtained from all study participants and/or their legal guardians.

## Results

### Association Analysis in the HSCR-DS Sample

SNP-genotyping on chromosome 21 was carried out in 26 Caucasian patients with DS and HSCR and their parents for 12,579 SNPs in chromosome 21. When calling genotypes with the K-means method for independent individuals, the number of M1 and M2 trios among trios with heterozygotes mother was significantly greater than the number of F1 and F2 trios among trios with heterozygotes father for all trios but one for which it was significantly lower (see Table S1). Therefore, we were able to infer a maternal non-disjunction for all trios but one displaying paternal non-disjunction.

1,065 SNPs (8.5%) were discarded because of Hardy Weinberg disequilibrium in parents. Additional 181 SNPs (1.5%) were discarded because children genotype calls performed using K-means for independent individuals and for trios data differed in more than 6 SNPs (25% of the SNPs). Therefore, 11,333 SNPs (90%) were tested for association with HSCR.

**Figure 1:**
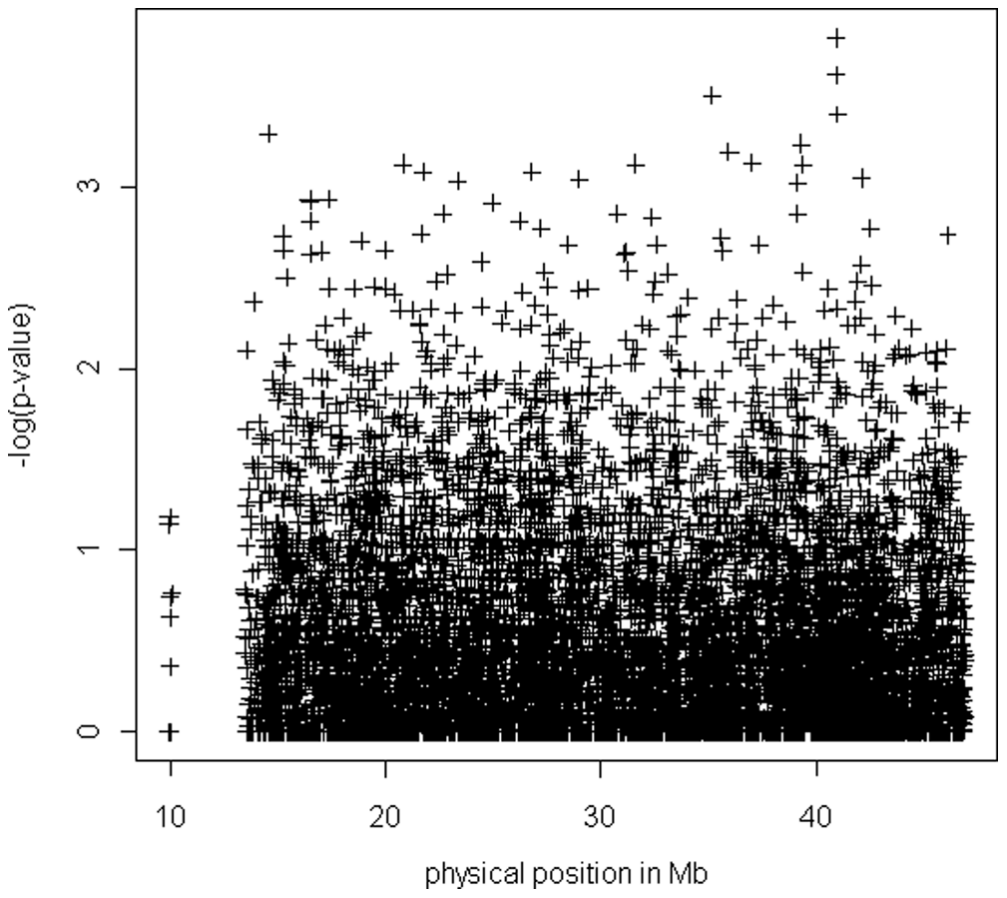
Chromosome-21-wide p-values for the HSCR-DS sample.

**Figure 2:**
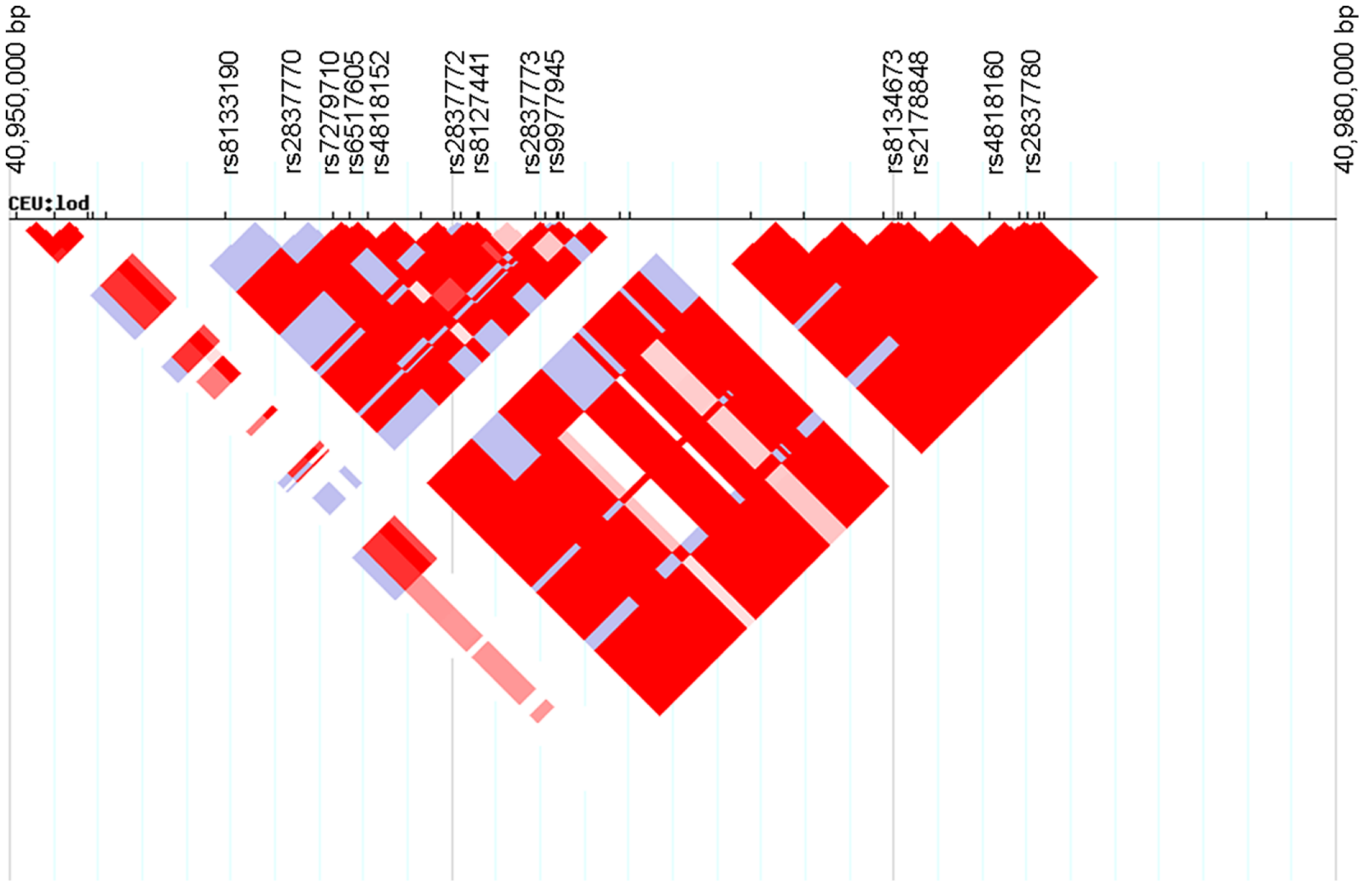
HapMap release 27 phase II+III, Feb09, on NCBI B36 assembly, linkage disequilibrium plot for CEU sample for the region spanning 40,950 kb to 40,980 kb on human chromosome 21.

As shown in Figure 1, top p-value signals were achieved by 2 SNPs in complete association except in one parent: rs2837770 and rs8134673, both located in intron 3 of *DSCAM*. Detailed genotypes are indicated in Table 1. Of note, 24 parents out of 52 were heterozygous for rs2837770 and 23 parents for rs8134673,. Nominal p-value for rs2837770 and rs8134673 were respectively p = 1.5×10^−4^ and p = 2.4×10^−4^ and after correction for multiple testing p = 0.02 and 0.04. Both SNPs co-localized to the same linkage disequilibrium block, spanning from 40,954 kb to 40,973 kb (hg18), and encompassing an exon-free region (Figure 2). Of note, we applied the trisomic transmission disequilibrium test to rs2837770 that we implemented using the function optim (method “L-BFGS-B”) in R 2.15.2 software and found very similar p-value (p = 0.00026). Within this region, 18 SNPs were successfully genotyped, and 7 of them were associated with nominal p<0.05 (Table 2). We thus focused on this region for the validation step.

**Table 1:** Genotype at rs2837770 for the 26 triads.

Triad number	CDJP	NDJP	Case
1	AG	AG	AAG
2	AG	GG	AGG
3	GG	AA	AAG
4	AA	GG	AGG
5	AG	AG	AAG
6	AG	AG	AAA
7	AG	GG	AGG
8	AG	AG	AAG
9	AA	GG	AGG
10	GG	GG	GGG
11	GG	AG	AGG
12	GG	GG	GGG
13	AA	AG	AAG
14	AA	AA	AAA
15	AG	AG	AAG
16	AG	AA	AAA
17	GG	GG	GGG
18	AG	AA	AAA
19	AG	AA	AAA
20	AG	AA	AAA
21	AG	GG	AGG
22	AG	GG	AGG
23	AG	AG	AAG
24	GG	GG	GGG
25	AA	AG	AAA
26	AG	GG	AGG

CDJP: Correctly disjoining parent.

NDJP: Non-disjoining parent.

**Table 2:** Association results for the SNPs in the region spanning 40954 kb to 40973 kb on chromosome 21 for the sample with DS and HSCR.

*Variant*	Position	Minor allele	Majorallele	MAF in HapMap CEU sample	MAF inpseudos	MAF incases	HWE	Case-pseudostest p-value	T	U	OR	CI 95%	
*rs8133190*	40954866	C	T	0.12	0.15	0.08	0.65	0.10	11	5	2.20	0.76	6.33
*rs2837770*	40956222	A	G	0.41	0.31	0.55	0.57	0.00015	7	26	0.27	0.12	0.62
*rs7279710*	40957328	T	C	0.12	0.14	0.09	0.65	0.20	6	10	0.60	0.22	1.65
*rs6517605*	40957682	C	T	0.57	0.47	0.63	0.86	0.04	11	23	0.48	0.23	0.98
*rs4818152*	40958090	T	C	0.13	0.22	0.13	0.66	0.16	9	16	0.56	0.25	1.27
*rs2837772*	40960044	T	C	0.31	0.31	0.24	0.85	0.48	17	12	1.42	0.68	2.97
rs8127441	40960563	G	A	0.39	0.31	0.58	0.34	0.0004	6	27	0.22	0.09	0.54
rs9984320	40960601	T	C	NA	0.47	0.64	0.86	0.01	14	27	0.52	0.27	0.99
rs2142126	40961286	G	C	0	0.03	0.01	0.89	1.00	1	2	0.50	0.05	5.51
rs2837773	40961880	C	A	0.42	0.46	0.35	0.52	0.19	20	11	1.82	0.87	3.79
rs9977945	40962128	T	C	0.12	0.15	0.08	0.65	0.10	11	5	2.20	0.76	6.33
rs9977484	40962357	G	C	NA	0.17	0.05	0.30	0.02	13	4	3.25	1.06	9.97
rs8130310	40962412	C	G	NA	0.1	0.13	0.65	0.64	9	7	1.29	0.48	3.45
rs2837774	40962525	C	T	NA	0.46	0.36	0.01	0.30	18	10	1.80	0.83	3.90
rs8134673	40970181	A	G	0.39	0.27	0.55	0.63	0.00024	6	28	0.21	0.09	0.52
*rs2178848*	40970468	A	G	0.5	0.47	0.33	0.08	0.03	21	10	2.10	0.99	4.46
rs4818160	40972146	T	C	0.5	0.47	0.56	0.99	0.29	24	17	1.41	0.76	2.63
rs2837780	40972828	C	T	0.3	0.28	0.31	0.03	0.65	10	12	0.83	0.36	1.93

MAF: minor allele frequency, HWE: Hardy Weinberg Equilibrium test p-value, T: number of minor alleles transmitted to the affected case from heterozygotes parents, U: number of minor alleles untransmitted to the affected case from heterozygotes parents, CI 95%: 95% confidence intervals for odds-ratio, OR: odds-ratio.

### Validation of the Association in the HSCR Non-syndromic Sample

We further tested the association between HSCR and all SNPs of the 19 kb-long region spanning from 40,954 kb to 40,973 kb using the isolated-HSCR sample, typed on either the *NspI* array (n = 125) and/or the *StyI* array (n = 128). For this sample, 5 SNPs were successfully genotyped, among which rs2837770 was the only one in common to our previous analysis (Table 3). We found that rs2837780 was strongly associated to HSCR in this validation sample (nominal p = 0.00065, p = 0.0032 after a Bonferroni correction for 5 SNPs). Of note, rs2837780 was not associated to HSCR in the HSCR-DS sample, but showed departure from Hardy-Weinberg disequilibrium (p = 0.03). This could be an indirect argument in favour of association with HSCR [18].

**Table 3:** Association results for the SNPs in the region spanning 40954 kb to 40973 kb for the isolated HSCR sample.

Variant	Position	Number of cases-pseudos pairs	Minor allele	Major allele	MAF in cases	MAF inpseudo-controls	p-value	T	U	OR	CI for OR
*rs2837770*	40956222	120	A	G	0.44	0.36	0.09	67	49	1.37	[0.95; 1.97]
*rs7279710*	40957328	124	T	C	0.12	0.14	0.48	26	32	0.81	[0.48; 1.37]
*rs9977945*	40962128	111	T	C	0.12	0.14	0.46	23	29	0.79	[0.45; 1.37]
rs2837774	40962525	122	C	T	0.42	0.48	0.17	52	67	0.78	[0.55; 1.11]
*rs2837780*	40972828	112	C	T	0.2	0.34	0.00065	28	58	0.49	[0.31; 0.76]

### Characterization of DSCAM Involvement

**Figure 3:**
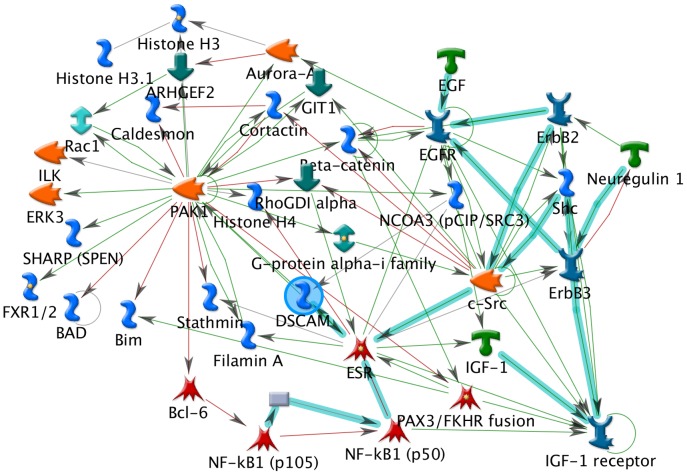
Network involving DSCAM in autonomous nervous system development. Using the MetaCore platform, this biological network was established after filtering DSCAM as network objects. DSCAM, the only protein subjected to the filters of expression, is encircled.

We then questioned systems biology protein networks on *DSCAM* gene. To this end, we allowed the platform to build network for nervous system development. As shown in Figure 3, we focused on DSCAM as a prioritized network object, using filters on brain, fetal brain and colon as tissue of expression (Figure 3). Careful analysis of the network unravelled the involvement of *neuregulin-1* (*NRG-1*), an HSCR gene [19], in the same pathway.

**Figure 4:**
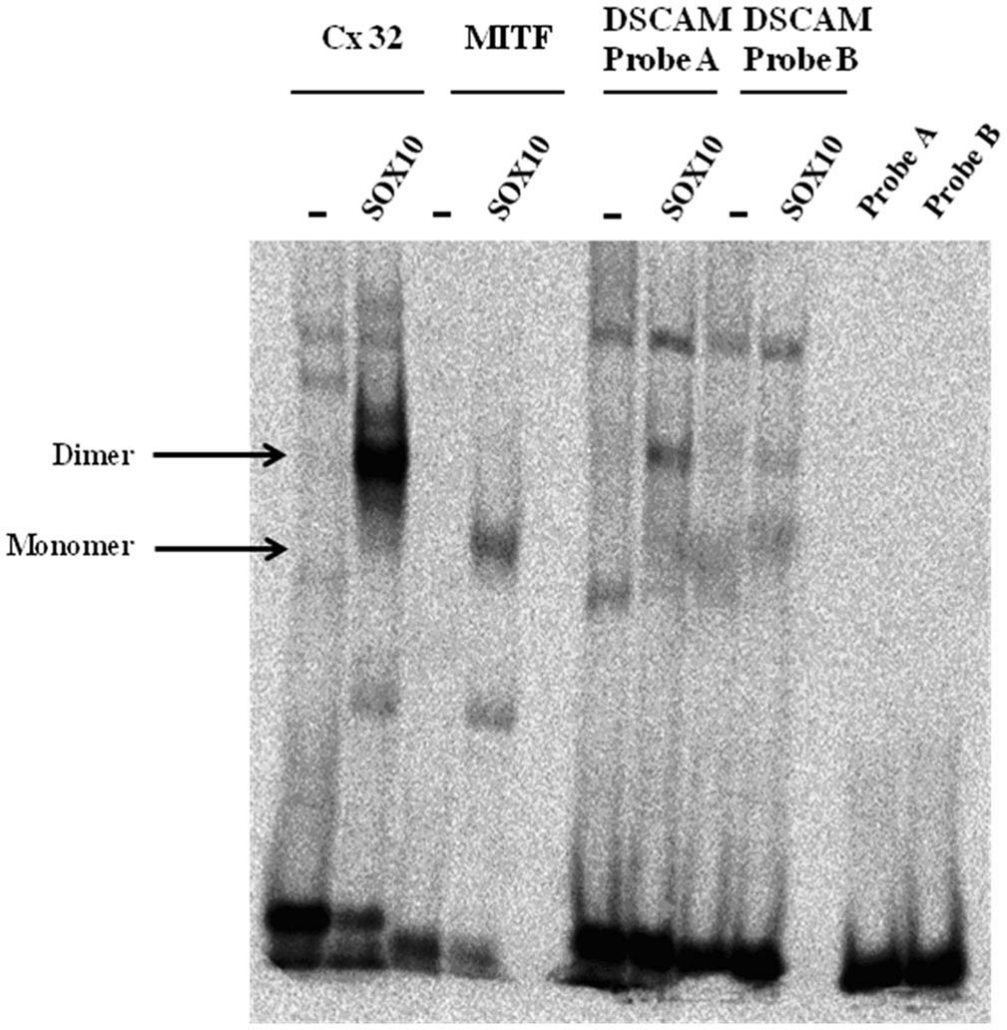
Electrophoretic mobility shift assays using the dimeric binding site from the *Cx32* (line 1 and 2), the monomeric binding site from the *MITF* (line 3 and 4) promoter regions, DSCAM probes containing the A allele from rs2037778 (probe A; line 6, 7 and 11) or the G allele (probe B; lines 8, 9 and 12), and empty pECE vector (−), or SOX10. The last two wells correspond to probes A and B alone.

To gain further insights into the functionality of the association results, we studied the 19 kb-long region spanning 40,954 kb to 40,973 kb in terms of conserved composite motif discovery for SOX10-binding sites. Indeed, SOX10 is a key transcriptional regulator of neural crest development, which also regulates *RET* gene. We found that the A allele of rs2837778 was part of a SOX10-binding site, unlike the G allele. Interestingly, we observed that the A allele at rs2837778 was in complete association with the T allele of rs2837780 in HapMap CEU population (1000genomes CEU low coverage). Other putative SOX10-binding sites were identified close by, one of them corresponding to a conserved binding site (both binding sites are underlined in the probe sequence provided in materials and methods). Interestingly both sites are pointing towards each other and separated by 9 pb, a configuration previously described as optimal for dimeric SOX10 binding [20]. Thus, we further challenged the possibility of SOX10 to effectively bind this sequence. By electrophoretic mobility shift assay, we detected a significant dimeric binding of SOX10 to a *DSCAM* fragment carrying the A allele whereas reduced binding was observed with the G allele (Figure 4).

## Discussion

For long, *DSCAM* has been regarded as an appealing candidate gene accounting for the increased prevalence of HSCR in patients with DS. *DSCAM* was shown to map to HSCR critical region [7] in patients with DS as well as to the genomic region associated with HSCR in a large Mennonite kindred [21]. However, its involvement in HSCR epidemiology had never been provided since then, as recently underlined by different studies, which aimed at discovering the genes involved in HSCR on chromosome 21 [5], [22]. Our results point to *DSCAM* as a predisposing locus to HSCR in patients with DS. We first identified two SNPs, rs2837770 and rs8134673 that co-localize to the same linkage disequilibrium block encompassing an exon-free region of 19 kb in length of the *DSCAM* gene, in association with HSCR in our HSCR-DS sample. This association was then replicated using an independent sample of isolated HSCR cases (without DS). Consistently, rs2837770 was recently shown to be associated to HSCR in a Chinese population [23]. We thus propose that the involvement of *DSCAM* in HSCR susceptibility will be encountered in most ethnical backgrounds.

Remarkably, a great variety of arguments from the literature converge to emphasize the relevance of *DSCAM* to HSCR. Beyond genetic analyses, the expression and the function of DSCAM are both relevant to HSCR. *In situ* hybridization analyses of the mouse *Dscam* gene revealed a broad expression pattern within the nervous system at the time of neuronal differentiation namely in the neural tube, cortex, hippocampus, medulla, spinal cord and most neural crest-derived tissues [24]. Furthermore, mice deficient for *Dscam* display a significant loss of pre-inspiratory neuron synchronicity and perinatal death, similar to congenital central hypoventilation syndrome (Ondine’s curse), in which patients are predisposed to HSCR [25]. Moreover, DSCAM is a receptor for netrin-1 [26], [27]. Netrin-mediated guidance is essential for the development of submucosal ganglia [28]. Mice mutant for deleted-in-colorectal-carcinoma (DCC), which is a netrin-1 dependence receptor, lack submucosal gut ganglia [29]. Vagal neural crest-derived precursors of the enteric nervous system colonize the bowel rostro-caudally within the enteric mesenchyme. Orthogonal secondary migrations, towards the mucosa, result in the formation of submucosal ganglia. This perpendicular migration is in part mediated by netrins that are known to be expressed in the mucosa of the fetal intestine [30]. Thus, a role of DSCAM in the secondary migration of neurons in the gut, being a RET-independent pathway, could explain why the *DSCAM* gene was not differentially expressed in the enteric nervous system of *Ret* mutant mice compared to controls [22]. Interestingly, the pathway analysis conducted herein reveals further links between *DSCAM* and HSCR. In particular, *NRG-1*, a gene in the network was shown to be associated to HSCR in a genome-wide analysis of a Chinese cohort [19], [31], and also in a Spanish cohort [32]. In fact, *NRG-1* is a ligand of *ErbB2* and *ErbB3*, which are essential for development of the sympathetic nervous system [33]. Both receptors have been localized in enteric neurons [34] and are known to activate estrogens receptors [35]. Of note, *DSCAM* expression is sensitive to estrogens via a clustering of 10 estrogens receptor binding sites in the same intron downstream the linkage disequilibrium where the SNPs associated to HSCR lie [36]. Estrogens have also been shown to regulate the major HSCR gene, *RET* [37].

DSCAM is also known as a member of the large family of cell-adhesion molecules. Interestingly, *L1CAM,* which is another gene from this family, predisposes to HSCR. Indeed, *L1CAM* mutations have been ascribed to a X-linked hydrocephaly syndrome (MIM) with predisposition to HSCR [38], [39], [40]. *L1CAM* is expressed in the enteric nervous system [22] and is required for chain migration of neural crest cells in the developing mouse gut [41]. An interaction between *L1CAM* and *SOX10,* a HSCR gene [42], was shown to significantly impair neural crest migration towards the developing gut [43]. Because SOX10, which is known as a key transcriptional regulator of neural crest development [42], [44] regulates *RET*, the major HSCR locus, via binding to very similar consensus sequences within *RET* intron 1, we examined the exon-free region of 19 kb for SOX10 regulation. Both *in silico* and *in vitro* analysis showed that allele A at rs2837778, a SNP in complete association to the most associated SNP in the isolated-HSCR sample, unravels a SOX10-binding site. Such regulatory mechanism would explain why both abnormal dosage and allelic differences could modify susceptibility to HSCR.

Association studies dealing with trisomic patients performed thus far not only never focused on HSCR but also never implied any chromosome 21-wide association study. Most studies involving patients with DS dealt with congenital heart defects, which is a frequent DS-associated phenotype and tested for association with specific genes, loci or gene pathways on chromosome 21. Locke et al [45] focused on genes involved in the folate metabolism using a large sample of trios for DS cases with congenital heart diseases and a control sample of trios for DS cases without congenital heart defect and their parents. Xu et al [13] proposed a trisomic transmission disequilibrium test and applied their method to a SNP located in *SH3BGR*, a gene expressed in fetal heart tissue and located in the DS critical region for congenital heart defect. Kerstann et al [46] performed an association study (case-control and a transmission disequilibrium test) to the region shown to be the minimal critical region for congenital heart defect on chromosome 21. None of these studies were conclusive. Therefore, our chromosome 21-scan study provides a new methodology to unravel the genetic determinism of other sub-phenotypes in DS patients. As a future step, it will be of interest to assess whether the most associated SNPs in *DSCAM* exhibit SNP frequencies in patients with DS but not with HSCR that are similar to the ones in the general population.

## Supporting Information

Table S1 **Number of F1 and F2 configurations (F1+F2) and M1 and M2 configurations (M1+M2) when calling genotypes with the K-means method for independent individuals.**
(DOC)
