# Supplementary material for: Chromosome 21 Scan in Down Syndrome Reveals DSCAM as a Predisposing Locus in Hirschsprung Disease
Source: PLoS One. 2013 May 6;8(5):e62519. doi: 10.1371/journal.pone.0062519 (PMC3646051; doi:10.1371/journal.pone.0062519)
Supplement: Table S1 — Number of F1 and F2 configurations (F1+F2) and M1 and M2 configurations (M1+M2) when calling genotypes with the K-means method for independent individuals. (DOC) [file pone.0062519.s001.doc]

**Supplemental material**

**Table S1: Number of F1 and F2 configurations (F1+F2) and M1 and M2 configurations (M1+M2) when calling genotypes with the K-means method for independent individuals**

| **Triad number** | **F1+F2** | **M1+M2** |
| --- | --- | --- |
| **1** | **514** | **94** |
| **2** | **147** | **79** |
| **3** | **403** | **71** |
| **4** | **174** | **87** |
| **5** | **465** | **79** |
| **6** | **447** | **63** |
| **7** | **428** | **47** |
| **8** | **135** | **50** |
| **9** | **439** | **88** |
| **10** | **407** | **85** |
| **11** | **78** | **207** |
| **12** | **265** | **89** |
| **13** | **157** | **72** |
| **14** | **197** | **70** |
| **15** | **144** | **103** |
| **16** | **510** | **82** |
| **17** | **151** | **68** |
| **18** | **406** | **54** |
| **19** | **641** | **56** |
| **20** | **899** | **59** |
| **21** | **181** | **88** |
| **22** | **411** | **127** |
| **23** | **170** | **102** |
| **24** | **118** | **75** |
| **25** | **816** | **79** |
| **26** | **281** | **88** |
